# Fertility, Migration and Acculturation (FEMINA): a research protocol for studying intersectional sexual and reproductive health inequalities

**DOI:** 10.1186/s12978-019-0795-5

**Published:** 2019-09-11

**Authors:** Violeta Alarcão, Miodraga Stefanovska-Petkovska, Ana Virgolino, Osvaldo Santos, Sofia Ribeiro, Andreia Costa, Paulo Nogueira, Patrícia M. Pascoal, Sónia Pintassilgo, Fernando Luís Machado

**Affiliations:** 1 Centro de Investigação e Estudos de Sociologia (CIES-IUL), Instituto Universitário de Lisboa (ISCTE-IUL), Av. das Forças Armadas, 1649-026 Lisboa, Portugal; 2 Instituto de Saúde Ambiental, Faculdade de Medicina, Universidade de Lisboa, Avenida Professor Egas Moniz, 1649-028 Lisboa, Portugal; 3 Instituto de Medicina Preventiva e Saúde Pública, Faculdade de Medicina, Universidade de Lisboa, Avenida Professor Egas Moniz, 1649-028 Lisboa, Portugal; 4 Escola Superior de Enfermagem de Lisboa, Lisboa, Portugal; 5 Laboratório de Biomatemática, Faculdade de Medicina, Universidade de Lisboa, Avenida Professor Egas Moniz, 1649-028 Lisboa, Portugal; 6 CICPSI, Faculdade de Psicologia, Universidade de Lisboa, Alameda da Universidade, 1649-013 Lisboa, Portugal; 7 Escola de Psicologia e Ciências da Vida, Universidade Lusófona de Humanidades e Tecnologias, Lisboa, Portugal

**Keywords:** Sexual and reproductive health inequalities, Intersectionality, Immigrants, Fertility, Acculturation, Survey, Qualitative research

## Abstract

**Background:**

The existing knowledge on the interplay between reproductive and sexual health, migration and acculturation is recent and inconsistent, particularly on the sociocultural motives and constraints regarding fertility. Therefore, sexual and reproductive health (SRH) surveys are needed to provide accurate and comparable indicators to identify and address SRH inequalities, with specific focus on under researched aspects, such as the interrelation between migration and gender. FEMINA (FErtility, MIgratioN and Acculturation) aims to investigate intersectional SRH inequalities among Cape Verdean immigrant and Portuguese native families and how they impact on fertility in Portugal. This study will use a comprehensive approach exploring simultaneously the components of SRH, namely regarding identities, perceptions and practices of both women and men among lay people and relevant experts and stakeholders. The project has three main goals: 1) to identify social determinants of SRH among Cape Verdean immigrant and Portuguese native men and women of reproductive age; 2) to gain understanding of the diversity of the sexual and reproductive experiences and expectations of Cape Verdean immigrant and Portuguese native men and women of reproductive age, considering the singularities of their migratory, social and family dynamics; and 3) to produce recommendations for policy makers, employers and service providers on how to better address the SRH needs of Portuguese-born and immigrant populations.

**Methods:**

The study will address these goals using a mixed methods approach, including: a cross-sectional telephone survey with a probabilistic sample of 600 Cape Verdean immigrant and 600 Portuguese native women and men (women aged 18 to 49 and men aged 18 to 54), residents of the Greater Lisbon Area; a qualitative research through in-depth interviews with a subsample of 30 Cape Verdean immigrants and 30 Portuguese native men and women; and a Delphi technique for finding consensus on good practices in SRH for the entire population with a special emphasis on immigrants, namely extra-EU migrants.

**Discussion:**

Data will be used to produce a comprehensive set of indicators to monitor SRH in Portugal, to foster a greater understanding of its specificities and challenges to policy and decision makers, and to provide targeted recommendations to promote inclusive and migrant sensitive SRH services.

## Plain English summary

Sexual health and reproductive health are equally important parts of personal health and development. For many years the focus of research on sexual health has concentrated mostly on issues such as the prevention of diseases, infections and unplanned pregnancies. Nowadays the focus of this research has expanded to include sexual and reproductive health rights that encompass our sexual health, gender equality and empowerment of women. However, despite the advancements being made, challenges in terms of the fulfillment of the diversity of sexual and reproductive health needs across life course and populations still exist. Migrant populations can be particularly vulnerable to sexual and reproductive health issues due to gender and socioeconomic inequalities, cultural and social norms around sexuality, and other social and structural factors. These include, among other things, how old is the migrant population, how the migrant population will adapt to the host culture, how well will it be able to navigate through the health system and linguistic barriers, and how all these factors will impact not only their fertility capacity and status, but also their achievement of sexual health. Thus, some researchers have considered that the process of postmigration cultural adjustment (i.e., acculturation) may induce a change in how individuals make decisions about important events such as when and whether to have a child. However proper evidence that establishes the link between migration, sexual health and reproductive decisions is still lacking. Therefore, there is a need to study how different generations and genders in immigrant families in Portugal perceive the concepts of family and sexuality based on the institutional and policy context that surround them. The FEMINA (FErtility, MIgratioN and Acculturation) study proposes to explore whether sexual and reproductive health inequalities impact on fertility among Cape Verdean immigrant and Portuguese native families in Portugal.

## Introduction

Sexual and reproductive health (SRH) constitutes a crucial part of general health and a central feature of human development [1]. The conceptualization of SRH has progressed from a focus on the prevention of ill-health, including sexual ill-health, unwanted pregnancies, sexually transmitted infections and sexual violence to a broader concept where sexual health is seen as a determinant for health and as a prerequisite for reproductive health [2]. Nowadays, SRH has evolved to cover other dimensions, entailing a focus on equity, gender equality and human rights, including sexual rights [3]. An example is the International Conference on Population and Development, which was held in Cairo, Egypt, in 1994, and its resulting Program of Action, which has been moving population policies and programs to the recognition that sexual health, including reproductive health, and sexual rights, as well as gender equality and women’s empowerment, are important ends in themselves and key to improving the quality of life for everyone [4, 5].

Significant positive achievements have been made over the past three decades in the legal and policy protection and recognition of women’s sexual and reproductive rights in Europe, ranging from the legalization of abortion to an increased protection against gender-based violence. However, significant inequalities and disparities in the enjoyment of sexual and reproductive rights continue to affect marginalized groups of women, including those belonging to ethnic minorities, undocumented migrants and asylum seekers, as well as those who are economically disadvantaged [6]. According to the World Health Organization (WHO), migrants represent one of the most vulnerable populations when it comes to SRH due to the growing complexity and heterogeneity of migration flows with impact in terms of the different health determinants, needs and vulnerabilities of the migrant populations [7].

Today, there are an estimated 214 million international migrants, 740 million internal migrants, with an unknown number of migrants in an irregular situation all over the world [7]. In 2011, 9.7% of the total European Union (EU) population consisted of foreign-born residents. During 2016, 4.3 million people emigrated to one of the EU-28 members states, and almost half of these were citizens of non-EU countries [8]. With regard to Portugal, in 2017, women of foreign nationality gave birth to about 10% of the total number of live births, despite the fact that the foreign population accounted for only 4.1% of the total population [9]. Another potential reason for the vulnerability of migrant populations is the multidimensional phenomenon of the feminization of migration. On one hand, migration can reaffirm structural barriers and gender inequality, with women migrants being concentrated in low skilled, low paid and often informal sectors. On the other hand, migration can also create opportunities for women’s economic empowerment and foster positive outcomes for gender equality [10]. This dual path of the dynamic of the migration-development nexus may impact women and men’s sexual health and reproductive decisions.

An in-depth look at the immigration numbers will reveal different types of migrating populations, but it will also indicate the importance of considering the collective health needs and implications of these population cohorts. For example, existing studies have demonstrated that fertility intensities tend to be high during the time immediately following migration among several migrant groups in Europe and the USA [11, 12]. In addition, a series of factors such as culture, education, integration, welfare, the age at migration and the reasons for migrating have been shown to affect migrant fertility behavior [13–18]. Notwithstanding, conclusive evidence on whether acculturation produces a shift in fertility behaviors of immigrants and on how individuals make decisions about vital events such as migration to another country as well as whether and when to have a child (or a second or a third), is still lacking. There is the need to study generational and gender changes related to practices and attitudes towards sexuality and sexual relationships among immigrants, and to conduct more comparative research to deepen our understanding of how ethnic minorities structure their families and intimate lives in different institutional and policy contexts [19].

Within the EU, migrants’ right to health care, in particular SRH care, is currently not ensured [20]. The scarce public policies and strategic frameworks addressing migrants’ health fail to address the several intertwined SRH areas, providing a narrow focus only on HIV screening and on perinatal care [20]. The broader conceptualization of sexual health and its linkages to reproductive health also seems to be less visible in the existing research in Portugal [21–23], though with some important contributions to the construction of immigration policies to promote social inclusion and well-being of migrant populations [24, 25]. The need for such actions is in compliance with the 2030 Agenda for Sustainable Development which includes a goal on gender equality and empowerment (Goal 5) as well as a goal on reduction on inequality within and among countries (Goal 10). The first provides the opportunity to empower women migrant workers, transnational mothering and mainstream gender and migration in legislative policy and framework; whilst the latter increases legal migration channels, and straightens gender inclusive social protection and gender-responsive labor migration governance [26]. Inclusive indicators to monitor SRH, to foster a greater understanding of its challenges and specificities, and to provide targeted recommendations to respect, protect and promote SRH and human rights for all populations are needed.

There have been several national surveys in Portugal that incorporated indicators of SRH. However significant gaps can be observed in the SRH data coming from these studies. The studies are presented in Table 1 and compared with the present study protocol. Five national Health Interview Surveys (HIS) have been conducted in 1987/88, 1995/96, 1998/99, 2005/06 [24] and 2014 [27]. However, information on contraception and on screenings for cervical and breast cancer became a regular component of this survey only from 2005/06 on (within the sections Family Planning/Reproductive Health and Preventive Care). Apart from HIS, nationally representative surveys collected only partial relevant information for SRH, such as the Health and Sexuality Survey conducted in 2007 [28] and the Fertility Survey developed in 2013 [29]. Regarding the socio-demographic profile of the participants, the majority of studies only provide generational and gender comparisons of the findings, with no specific focus on immigrant groups. An exception was a report using data from the HIS 2005/06 with the immigrant population in Portugal [30]. As seen in Table 1, data intersecting SRH outcomes with immigration/ethnicity and gender are lacking in Portugal.

**Table 1:** Sexual and reproductive health data from national surveys

	National health interview survey^a^	Health and sexuality survey	Fertility survey^b^	FEMINA
Year	2005/06 and 2014	2007	2013	2020
Study design	cross-sectional	cross-sectional	cross-sectional	cross-sectional
Focus of the study	to characterize the resident population aged 15 years or over in three domains: health status, health care, and health determinants	to analyze the relationships between sexual behavior and risk behavior associated with the transmission of HIV	to contribute to a more thorough knowledge of fertility in Portugal	to study intersectional sexual and reproductive health inequalities
Survey organization	Instituto Nacional de Estatística / Instituto Nacional de Saúde Doutor Ricardo Jorge	Instituto de Ciências Sociais	Instituto Nacional de Estatística / Fundação Francisco Manuel dos Santos	Instituto Universitário de Lisboa / Instituto de Saúde Ambiental
SRH indicators				
Fertility and reproductive history	Yes	Yes	Yes	Yes
Sexuality and sexual relationships	No	Yes	No	Yes
Women’s reproductive health	Yes	Yes	Yes	Yes
Men’s reproductive health	No	No	Yes	Yes
Sexual dysfunctions	No	Yes	No	Yes
Sexually transmitted infections	No	Yes	No	Yes
Does the study intersect sexual and reproductive health outcomes with immigration/ethnicity and gender?	No	No	No	Yes

^a^There are five National Health Interview Surveys (1987/88, 1995/96, 1998/99, 2005/06 and 2014. However, sexual and reproductive health relevant data is available only for the last two waves

^b^Another Fertility Survey will be conducted in 2019

Moreover, health surveys tend to be less suited to be representative of relevant sub-groups, such as migrant or transient populations, as their samples usually do not include enough participants to reflect the wide variability of the diverse immigrant population and available data are not disaggregated by important explanatory variables and social determinants of the migrants health. Finally, although previous studies have highlighted the importance of understanding the individual, the provider and the system’s challenges that immigrant men and women face when navigating the healthcare system, there is no research in Portugal that contributes to this discussion and to formulating tailored strategies that serve these groups [31–33]. Therefore, the use of data that overcome these limitations has to be encouraged [34].

Following the comprehensive approach of the Fertility, Contraception, and Sexual Dysfunction study (FECOND), a population-based probability telephone survey conducted in France in 2010 comprising 8645 respondents aged 15–49 years [35], this study will analyze contemporary practices, specificities and challenges for SRH in Portugal, additionally accounting for the increasing cultural diversity of the population and creating an opportunity for fostering health equity. In fact, previous research indicated not only that demographic and social change in Portugal have provided new opportunities specially for women and for their sexual lifestyles, but also that further research should explore ethnically diverse population and examine how changes in traditional/cultural norms about sex and sexuality affect SRH [36]. This study will serve as a scientific basis for both broad-based and targeted community initiatives for the promotion of sexual health.

The scope of the proposed research underlines the relevance of the knowledge produced by social sciences (in particular sociology) to understand the social phenomena such as fertility and sexuality [37]. Intersectionality is a theoretical framework for understanding how multiple social identities such as race, gender, sexual orientation, socioeconomic status, and disability interact at the micro level of individual experience to reflect linked systems of privilege and oppression (i.e., racism, sexism, heterosexism, classism) at the macro social structural level [38]. The explicit theorization and greater application of intersectionality within population health research has the potential to improve researchers’ collective ability to more specifically document inequalities within different groups, and to study the potential individual-level and group-level observed inequalities. It opens up the potential for examination of relevant questions regarding interactions between dimensions of oppression or privilege. [39].

### Study purpose

Although previous research has explored separately the issues of migration, gender and sexual health, including fertility care, their interactions remain under-explored and under-theorized [40]. The main focus of the available scientific evidence is on comparing migrant with native fertility rates, disregarding the potential of migrant fertility behavior as a point to evaluate the impact of a number of migration specific integration indicators [41], especially regarding the sensitizing the healthcare (both on system and provider level) to increase effective utilization by immigrant population, and to develop actions to promote the achievement of sexual health – physical, emotional, mental and social well-being related to sexuality for the entire population [3].

The purpose of this study is to develop a comprehensive research approach of the components of SRH related identities, perceptions and practices of both women and men among lay people (Cape Verdean immigrants and Portuguese natives) and key experts and stakeholders. FEMINA (FErtility, MIgratioN and Acculturation) will entail two main approaches. The first is based on the model of gendered sexuality over the life course, which posits that sexual beliefs and behaviors are the result of a lifelong accumulation of (dis)advantageous experiences and of the adoption/rejection of sexual scripts, within socio-historical contexts [42]. This framework helps explaining the coexistence of differences and similarities among individuals and holds particular promise for studying lifelong aspects of sexuality. The second approach deals with the complex factors that influence experiences of migration [43, 44], in particularly among the Cape Verdean communities living in Portugal [45–49]. Cape Verdeans living in Portugal constitute the second large group of foreigners legally residing in the country, representing in 2017 8.3% of the foreign population in Portugal. The majority of them are concentrated in the Greater Lisbon area [9].

### Research questions, goals and expected outcomes

**Fig. 1:**
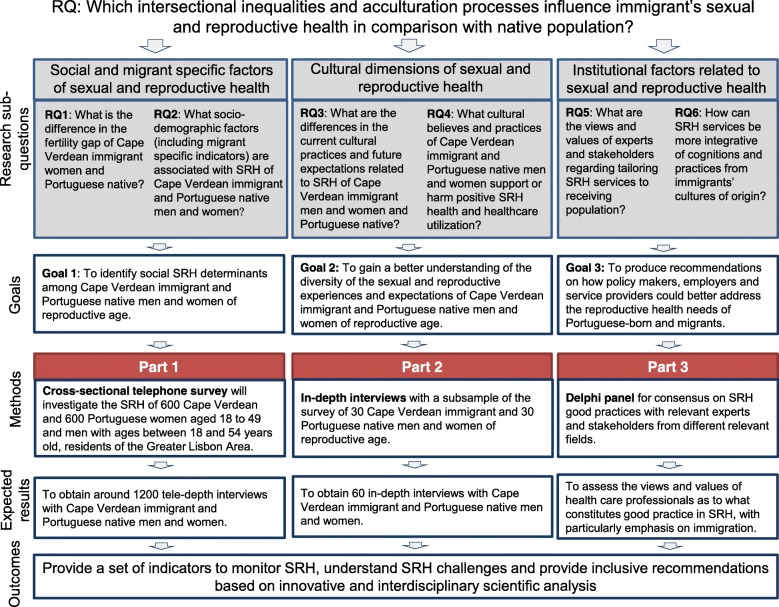
Research questions, goals and expected outcomes

The overarching research question (RQ) is: Which intersectional inequalities and acculturation processes influence immigrant’s sexual and reproductive health in comparison with native population in Portugal? The research question was addressed through the prism of three main research perspectives: [1] Social and migrant specific factors of SRH, [2] Cultural dimensions of SRH and [3] Institutional factors related to SRH, including access to healthcare, possible biases in the delivery of healthcare, and the quality of healthcare. These were broken down into six research sub-questions which produced three main study components and three research goals (Fig. 1).

## Methods

### Study setting and design

The existing literature highlights the existence of various forms of expression of the overall health and of SRH in particular, namely in terms of how individual identities, perceptions and practices are situated in relation to socio-economic factors and cultural contexts. Therefore, to answer the outlined research questions and address the specified goals, a multi-method research approach was adopted to explore both between and within group differences. It employs individual- and family-level quantitative and qualitative data collection on SRH and related relevant factors.

More specifically, for Part 1 of the study, a cross-sectional telephone survey will investigate the SRH of approximately 600 Cape Verdean immigrant and 600 Portuguese native men and women living in the Greater Lisbon area.

Part 2 aims to qualitatively handle the diversity of SRH-related beliefs and practices in the two populations under study. It is designed as a qualitative study consisting of in-depth interviews with a subsample of the survey of 30 Cape Verdean immigrant and 30 Portuguese native men and women (15 participants for each gender).

Finally, Part 3 of the study aims to establish consensus on good practices in the SRH field in Portugal, using a Delphi panel. For this purpose, experts and stakeholders will be drawn from different relevant fields (Academia, Nongovernmental Organizations, Policy­making, Health Care Practice and Civil Society Organizations).

### Sampling

**Fig. 2:**
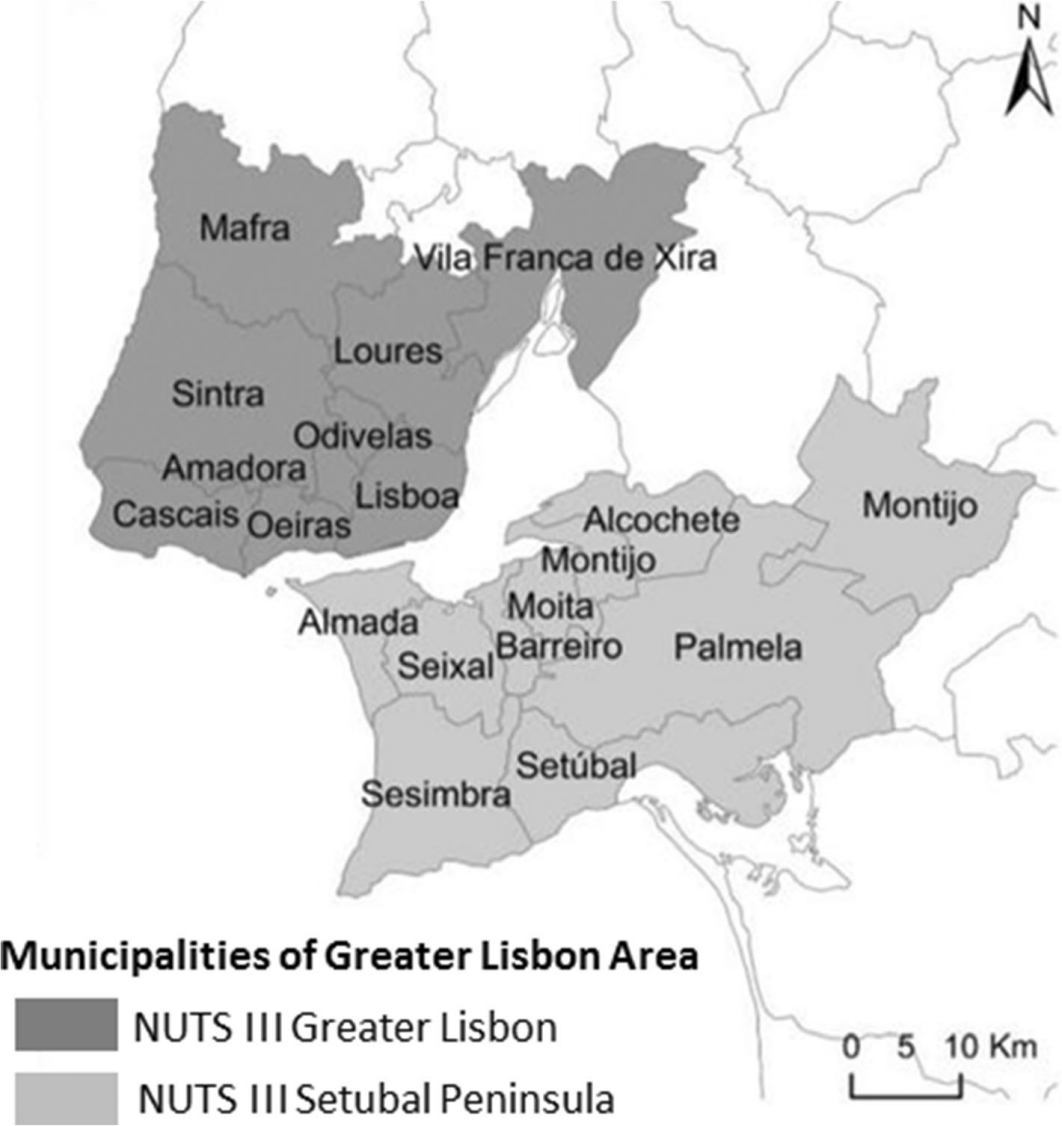
Presentation of the Greater Lisbon Area where data will be collected, adaptation from Louro & Marques da Costa [53]

For the purpose of the Part 1 of the study, a probabilistic sample of Portuguese native and Cape Verdean immigrant population will be selected through multistage sampling. The National Health Registry will be used as a sample frame, in collaboration with the Portuguese Central Administration of the Health System (CAHS). Since the CAHS has a central system of information which receives all data introduced in all primary health care centers (PHCC), this will enable the acquisition of data by sex, age, country of birth, and contact information, assuring that the participation will be independent of the regular use of the NHS [50]. These facilities are important since they are part of the government health care and constitute the first and most important portals for care of both domestic and immigrant populations [51, 52]. In the first step, ten PHCC will be randomly selected from the list of all PHCC of Great Lisbon area (Fig. 2). In the second step, individuals will be randomly selected within each health unit in accordance with inclusion criteria that will be specified later.

For Part 2 of this research, face-to-face in-depth interviews will be conducted with a sub-sample randomly selected of 30 Cape Verdean immigrant and 30 Portuguese native men and women among the participants of the cross-sectional telephone survey who have consented to remain in the study. In order to maximize the variation related to the attitudes and practices towards family life, fertility and parenting, participants will be classified and then randomly selected from the emergent typology.

For Part 3 of this study, experts in the Delphi panel will be selected by intentional sampling, based on their expertise in the areas of: SRH, general practice, and gender inequalities. Experts and relevant stakeholders with heterogeneous expertise, including both the social and health sciences, from research to policy action, will be drawn from Academia, Nongovernmental Organizations, Policy­makers, Health Care Practitioners and Civil Society Organizations in order to obtain a wider contribution from different scopes. Their identification will be done through literature review and with the collaboration of the research team and consultants. Attention will be given to experts and stakeholders with comprehensive knowledge of immigrant populations. A snowball sampling procedure will be used also to identify experts and stakeholders (initial experts and stakeholders identifying additional experts and stakeholders).

### Inclusion and exclusion criteria

For Part 1 and Part 2 of the study, the following inclusion criteria will be applied (Table 2): a) age – between 18 and 49 years old in the case of women (i.e. adult childbearing age) and between 18 and 54 in the case of men (i.e. when it’s more likely that they have or will have children); b) born in Portugal or in Cape Verde; c) both parents born in Portugal (for those born in Portugal) or both parents born in Cape Verde (for those born in Cape Verde); d) able to give informed consent to participate in the research. Each selected participant will be contacted by phone call to verify their eligibility. Exclusion criteria include: a) living in institutionalized households or collective residences; b) living in Portugal for less than 1 year; c) impossible to contact by telephone; d) persons who cannot understand and/or answer to the survey questions.

**Table 2:** Survey inclusion and exclusion criteria

Inclusion criteria	Exclusion criteria
1. Age – women aged between 18 and 49 years old and men between 18 and 54	1. People living in institutionalized households or collective residences:
	• hospitalized
	• incarcerated
2. Born in Portugal or in Cape Verde	2. Living in Portugal for less than 1 year
3. Both parents born in Cape Verde (for those born in Cape Verde) or both parents born in Portugal (for those born in Portugal)	3. Persons who are not reachable by telephone
	• without a valid telephone number
	• not reachable after seven attempts
4. Able to give consent to participate	4. Persons who cannot understand and/or answer the questionnaire:
	• due to a psychiatric condition
	• due to a severe hearing impairment
	• due to cognitive impairment

For Part 3 of the study, inclusion criteria will be: i) experts with knowledge on multiple dimensions of SRH and SRH inequalities; ii) stakeholders with the ability to influence policy in SRH at different sectors (public sector, private sector and civil society).

### Sample size estimation

In accordance to previously published relevant research procedures, a list of 1200 Cape Verdean immigrants and 1200 Portuguese natives (both genders) will be needed to account for 50% rate of exclusions and refusals [50]. In order to calculate sample size per group (Cape Verdean men, Cape Verdean women, Portuguese men and Portuguese women), we took into consideration the primary endpoint: decrease in birth rate, and used the formula presented in Noordzij et al. [54] for a binary outcome and equal sample size in both groups (p.1390). In line with data available from Carrilho & Craveiro [55], a total sample size for each group of at least 189 participants will be needed to detect a difference between two groups/percentages of 0.12 (i.e., difference between decrease in birth rates between 2001 and 2013: − 17% and − 29% for Cape Verdeans and Portuguese living in Portugal, respectively) with a statistical power of (1 - β = 0.8) and a significance level (alpha) of α = 0.05 (two-tailed). Considering maximum dropouts of 50%, the minimum sample size for each group will be approximately of 300 (600 Cape Verdean immigrant men and women and 600 Portuguese native men and women).

The qualitative research component (part 2 of the study) will be executed with a sub-sample of around 30 Cape Verdean immigrant men and women and 30 Portuguese native men and women. The proposed number will ensure (through a cluster analysis with data previously collected in Part 1) the creation of socially diverse groups with regard to key variables under study, beyond gender and country of birth, i.e.: age, level of education, occupational status, income, relationship status, nationality and residence time ratio in Portugal (in the case of women and men born in Cape Verde), typology of intention to have children, according to the existence of children (people who have children and intend to have more children; people who do not have children but intend to have; people who have children and do not intend to have more; people who do not have children and do not intend to have; and people who, whether or not having children, don’t know if they intend to have (more) children), facilitators and barrier to fertility, attitudes towards sexual and reproductive rights. The number of cases retained for analysis will follow two-fold criteria of empirical and theoretical saturation [56].

Finally, the number of panelists for the Delphi panel in Part 3 will be (at minimum) 75, dependent on the adequate mapping.

### Participants recruitment

Firstly, for study 1, approximately 2 weeks before starting data collection, invitation letters will be mailed to the sampled individuals notifying them that they will receive a telephone call. Using letter to forewarn individuals of a future telephone call increases the perceived legitimacy of the survey and has the potential to improve response rates [57, 58]. Additionally, the letter serves to clearly explain the purpose and nature of the study and also to provide the contact details for the lead researcher. Secondly, individuals will be contacted by telephone to verify their eligibility and confirm their willingness to participate. Both Cape Verdean immigrant and Portuguese native men and women who meet the inclusion criteria will be informed on the following aspects of the study: aims of the study, the way they were selected (random sampling, from the list of the healthcare unit), required voluntary engagement in the study, guarantee that the data will be anonymous and confidential and that the respondent can decline to answer any questions. The participants in the Part 1 – Sexual and reproductive health survey – will be asked for their permission to be identified and sampled for the Part 2 – Qualitative study. For the purpose of the Part 3 – Delphi panel –, the experts and stakeholders will be contacted by telephone, letter and/or e-mail. Their participation will be voluntary and will be preceded by informed consent collection.

### Data collection

Sexuality and gender interactions are complex phenomena. For that reason, methodological approaches combining multiple methods are indicated as ideal. Mixed methods will be used, as they are expected to produce a richer set of evidence on the understanding of sexuality, intimacy and reproduction in everyday life, through the dialectics between inductive and deductive theoretical developments (Pearce 2012). For the purpose of this research, data collection will involve the use of a sexual and reproductive health survey with Cape Verdean and Portuguese men and women of reproductive age to address Goal 1; a qualitative study among Cape Verdean and Portuguese men and women of reproductive age to address Goal 2; and a Delphi study among SRH experts and stakeholders to address Goal 3.

#### Part 1 – Sexual and reproductive health survey of Cape Verdean and Portuguese men and women of reproductive age

The sexual and reproductive health survey of Cape Verdean and Portuguese men and women of reproductive age will be conducted by telephone and recorded on computers (not audio-recorded), a process designated Computer-Assisted Telephone Interviewing (CATI). The expected average length of each interview will be 40 min. The data collection instrument will be only available in Portuguese language, which is also the official language of Cape Verde. However, at least one of the interviewers will speak Cape Verdean Creole/Cape Verdean language, in order to facilitate the data collection process with any individual who is less fluent in Portuguese. All tele-depth interviews will be performed by interviewers with background in sociology, psychology or similar relevant field, as well as previous research experience. All interviewers will undergo specific training on the topics under study, and will follow good practices and guidelines for sexuality-related surveys [59]. They will be continuously supervised by a member of the research team. Information will be collected on topics that are related to different aspects of SRH of participants. The procedures that will be implemented will be an adaptation of the FECOND study, while the methodology and instruments will be from the National Fertility Study conducted in 2013 [27], in order to allow for future comparisons. Finally, participants will be asked for their informed consent to be interviewed and for their permission to be sampled for the qualitative study of this project.

#### Part 2 – Qualitative study among Cape Verdean and Portuguese men and women of reproductive age

The qualitative survey of Cape Verdean immigrant and Portuguese native men and women of reproductive age will be preceded by the previously outlined sexual and reproductive health survey. This strategy will help to overcome the challenge of the sensitivity of this topic in face-to-face interviews. A sub-sample of randomly selected 30 Cape Verdean immigrant and 30 Portuguese native men and women who had given their consent to participate in this component of the study will be included in the in-depth interviews (same inclusion and exclusion criteria). Data collection will take place in a private room of the Primary health care centers to which participants belong to. It is assumed that the nature of the setting and familiarity with the environment will make it socially acceptable to discuss these issues. Interviews will be audio recorded and fully transcribed.

#### Part 3 – Delphi study among sexual and reproductive health experts and stakeholders

The aim of this study component is to identify what constitutes good practices in SRH and rights. For this purpose, a consensus construction design through a Delphi panel process will be followed with experts and stakeholders from different fields (Academia, Non-Government Organizations, Politicians, Clinical Practitioners and Civil Society Organizations) to assure the integration of indicators from multiple areas of concern and dimensions. This will enable the production of recommendations aimed at reducing sexual and reproductive inequalities. The Delphi survey will be delivered by *LimeSurvey* (or similar internet-based survey program), which will increase easing data entry, responses and analysis.

### Measures

#### Part 1 – Sexual and reproductive health survey of Cape Verdean and Portuguese men and women of reproductive age

Migration and childbearing are important life-course decisions which must be studied from a life-long perspective [41, 60]. Following a biographical logic, enabling to take into account complex relationships linking sexual and reproductive individuals’ life events [61], the tele-in-depth interviews will collect data on the following issues: marital and sexual history, attitudes and practices toward family life, fertility and fertility intentions, reproductive biography, support for infertility, contraceptive biography, use of health services relevant to SRH and evaluation of health professionals’ care, individual and family socio-demographic, migration/ethnicity and acculturation information.

More specifically, the survey will be used to collect data that will allow the exploration on the link between pregnancy intentions and contraceptive behaviors. Respondents will be asked to describe their reproductive history by providing detailed information on each of their pregnancies including the outcome (live births, elective abortions, miscarriages, ectopic pregnancies, therapeutic abortions, and stillbirths), the end date, pregnancy duration, birth delivery characteristics and evaluation of childbirth experience, postpartum follow-up of women’s health, partner relationship at the time of conception (stable, unstable starting, or breaking up) and their financial situation at the time of the conception and currently (no problems or difficult). For each pregnancy, respondents will be asked about their use of contraception in the month of conception and the reasons for non-use.

#### Part 2 – Qualitative study among Portuguese and Cape Verdean men and women of reproductive age

The Biographic-Narrative Interpretive Method [62] will be used to explore lay people representations of SRH: sexual and reproductive rights, SRH care, sexual satisfaction, sexual autonomy, fertility, parenthood, contraception. The notion of fixed and normative sexual and gender identities will be questioned and the complex factors that influence immigration and migration experiences explored, using Carpenter’s (2010) comprehensive framework to explore gendered sexuality over the life course. Regarding Cape Verdean participants, the identity and cultural diversity will be explored through the processes of integration and acculturation.

#### Part 3 – Delphi study among sexual and reproductive health experts and stakeholders

The Delphi process, with a minimum of three rounds which is considered sufficient to obtain a high level of agreement [63], will be organized with respect of the following:

1. In a previous qualitative phase of the Delphi panel, the indicators about views, values, barriers and good practices will be generated using a World Café methodology [64]. This will allow the generation of inputs, knowledge sharing, stimulation of innovative thinking and exploration of action possibilities.
2. After the identification of areas of concern, key dimensions and indicators, in the first round and for each indicator with the following statement “*This indicator is relevant to the evaluation of Sexual and Reproductive Health in Portugal*”, the experts and stakeholders will be asked to rank their agreement or disagreement with each item of the built-up list. All responses will be recorded on a 5-point Likert scale, where “I strongly agree” (SA) and “I agree” (A) indicates agreement, and “I strongly disagree” (SD) and “I disagree” (D) indicates disagreement. Panelists will be able to indicate “I neither agree nor disagree” (NAD) and a space for insert free-text comments will also be available. The number of agreements and disagreements will be calculated by expressing the participants answers in percentages per indicator. Indicators with more than 50% of SA and at the same time no more than 1/3 (33.3%) of SD and D responses will be immediately approved. On the contrary, indicators with more than 50% of SD or D will be immediately rejected by *absolute majority*.
3. For the second round, panelists will be presented with the results of the first round and asked to reconsider their answer regarding the group opinion. Also, the indicators that did not reach agreement will be included for reevaluation with respect to the information provided and with a view to contributing to greater agreement. The rules for approval and rejection by absolute majority will be kept. Additionally, to allow for an agreement on the selection of a high number of indicators, each indicator receiving more than 75% of SA and A will be approved by *qualified majority*.
4. For the third round, the same procedure will be applied and the group agreement will be determined by *qualified majority* maintaining the same rule of *absolute majority* for rejection.
5. All opinions will be kept confidential throughout the whole process.

### Data analysis

Statistical packages IBM SPSS® version 25 (or higher, at the time of the analyses) will be used to perform quantitative data analyses: univariate (central tendency and dispersion measures), bivariate (t-test, ANOVA, chi-squared or other non-parametric tests) and multivariate methods (linear and logistic regression). The two samples will be compared and regression models will be used to identify the factors associated with the different SRH outcomes (Part 1). For the qualitative data, the recorded voice of each in-depth interview will be transcribed integrally and labeled according to the type of information. Data analysis will follow a grounded-theory approach followed by an inductive analytical process. Qualitative data analysis will be carried out using MaxQda (or similar software). In part 3, statistical techniques usually used in Delphi studies will be applied to the responses given by the panel across rounds to describe the level of group agreement for each indicator [65]. Multivariate analyses of variance (MANOVA) will be performed to test the groups’ opinion variance across the type of panelist (experts vs. stakeholders) and across the different sectors (academia, non-government organizations, policy makers, clinical practitioners and civil society organizations).

After the completion of the three research components, the data will be integrated and analyzed in accordance to the specified project goals. Global data analysis will integrate intersectionality with multilevel analyses of the different contexts and variables. As a mixed-methods study, it will include the possibility for triangulation across quantitative and qualitative results to identify sites of concordance or divergence. Data will be compared with national and international landmark surveys: Portuguese Fertility Survey 2013 [29], United Kingdom National Surveys of Sexual Attitudes and Lifestyles (NATSAL-1, NATSAL-2 and NATSAL-3) [66] and France National Surveys - the FECOND study [35] and the Analyse des Comportements Sexuels en France (ACSF) [61].

## Discussion and conclusion

Immigration and migrant communities represent an inseparable part of contemporary societies, impacting and diversifying all spheres of its political, social, cultural and economic life [67, 68]. However, despite the rising awareness for the need to establish coordination between national and local level response in integrating the migrant populations, the existing literature lacks conclusive evidence on the role of acculturation on immigrant’s behaviors. In the area of interest of this project, there is insufficient research evidence on immigrants’ fertility related decision-making determinants, and their acculturation in the receiving society. Therefore, innovative research projects are essential to investigate cultural and social norms around sexuality and to conduct more truly comparative research to deepen our understanding of how sexual health and reproductive health are linked in different institutional and policy settings [19]. This project will build the research capacity in the area of SRH inequalities through the exchange of viewpoints of both women and men among lay people and relevant experts and stakeholders.

This study has some limitations that deserve discussion. First, this is a small-scale study. So, the results will not be generalizable for the Portuguese population nor for other migrant populations in Portugal. Second, the audio recording of the interviews may increase intrusiveness, with particular emphasis on topics concerning the SRH [37]. Finally, as the study concerns self-reported SRH data, it is worth to consider that social desirability bias may be problematic due to different expectations about social acceptable behavior [69].

Nevertheless, this study proposes a novel comprehensive analytical approach to contemporary issues of SRH by relocating the various events within individuals’ life trajectories and examining them from the point of view of the various agents involved (men and women, health professionals, civil society organizations and politicians). To our knowledge, none of the previously published studies have sought consensus on good practices in the SRH services field in Portugal [24, 27–29, 31–33]. At an international level, the few existing studies focus mainly on clinical guidelines. The results of this study can provide policy- and decision-makers with a set of indicators to monitor SRH and a greater understanding of SRH challenges that will provide directions on designing future interventions targeting the whole population as well as specific population groups. Furthermore, it is expected that the findings from FEMINA will benefit the scientific communities, clinical organizations and policymakers (both local and national) thereby having a larger impact on SRH interventions among population of reproductive age in Portugal, promoting equality in SRH and also among migrant’ populations.

## Data Availability

Not applicable.

## Abbreviations

ACSF: *Analyse des Comportements Sexuels en France* | Analysis of sexual behavior in France
CAHS: Central Administration of the Health System
CATI: Computer-assisted telephone interviewing
EU: European Union
FECOND: Fertility, Contraception, and Sexual Dysfunction
FEMINA: FErtility, MIgratioN and Acculturation
HIS: Health Interview Survey
HIV: Human immunodeficiency virus
IC: Informed consent
NATSAL: National Surveys of Sexual Attitudes and Lifestyles
PHCC: Primary health care centers
SRH: Sexual and reproductive health
WHO: World Health Organization
